# An Innovative Context-Based Crystal-Growth Activity Space Method for Environmental Exposure Assessment: A Study Using GIS and GPS Trajectory Data Collected in Chicago

**DOI:** 10.3390/ijerph15040703

**Published:** 2018-04-09

**Authors:** Jue Wang, Mei-Po Kwan, Yanwei Chai

**Affiliations:** 1 Department of Geography and Geographic Information Science, Natural History Building, 1301 W Green Street University of Illinois at Urbana-Champaign, Urbana, IL 61801, USA; kingjue.w@gmail.com (J.W.); mpk654@gmail.com (M.-P.K.); 2 Department of Urban and Economic Geography, College of Urban and Environmental Sciences, Peking University, Beijing 100871, China

**Keywords:** GIS, GPS, activity space, environmental exposure, the uncertain geographic context problem

## Abstract

Scholars in the fields of health geography, urban planning, and transportation studies have long attempted to understand the relationships among human movement, environmental context, and accessibility. One fundamental question for this research area is how to measure individual activity space, which is an indicator of where and how people have contact with their social and physical environments. Conventionally, standard deviational ellipses, road network buffers, minimum convex polygons, and kernel density surfaces have been used to represent people’s activity space, but they all have shortcomings. Inconsistent findings of the effects of environmental exposures on health behaviors/outcomes suggest that the reliability of existing studies may be affected by the uncertain geographic context problem (UGCoP). This paper proposes the context-based crystal-growth activity space as an innovative method for generating individual activity space based on both GPS trajectories and the environmental context. This method not only considers people’s actual daily activity patterns based on GPS tracks but also takes into account the environmental context which either constrains or encourages people’s daily activity. Using GPS trajectory data collected in Chicago, the results indicate that the proposed new method generates more reasonable activity space when compared to other existing methods. This can help mitigate the UGCoP in environmental health studies.

## 1. Introduction

In the fields of epidemiology and health geography, understanding environmental exposure is a nontrivial issue involving the investigation of environmental effects on human health. Researchers have examined the relationships among human movement, environmental context, and health outcomes over the past decades. Abundant research has shown that physical activity, tobacco use, obesity, mental health and many other health behaviors or issues are related to environmental exposure [1,2,3,4]. For instance, it has been found that the built environment influences physical activity and health [5,6]—e.g., obesity is more prevalent in areas that lack physical activity facilities [7] or are unfriendly to walking [8,9]. One of the fundamental questions in this research area is how to measure environmental exposure.

Despite the existence of many methods, the residential neighborhood is predominantly utilized as the contextual unit for environmental exposure measurement. It is often represented by administration areas, such as census tracts and postal units, because of the availability and easy access to routine administrative data. The readily available spatial delineations of administrative areas and the lack of detailed mobility data are other reasons for the popularity of administrative areas in environmental health research. With the help of advanced geospatial technologies (e.g., geographic information systems [GIS] and global positioning system [GPS]), there has been a methodological shift in health research, moving from using fixed administrative areas as contextual units to ego-centered definitions [10,11,12]. An ego-centered neighborhood is usually represented by a buffer area centered on an individual’s home with a given threshold of specific distance or travel time [13], which may reflect more accurately the exposure area rather than administrative units. Due to the ongoing debate as to the best way to define geographic context [14,15,16,17] and the availability of activity diary and GPS tracking data, many researchers have now adopted the idea that the residential neighborhood can only partially capture people’s exposure to environmental context, and daily activities that take place at other locations also contribute to their environmental exposures [10,18,19]. The shift from a static measuring approach to a dynamic one has inspired researchers to explore and develop exposure assessment methods using individual GPS tracking data [20,21,22]. 

Although there are many ways to measure environmental exposure, activity space based on GPS tracking data (movement data) appears to be a promising way to assess the environment utilized by individuals, and to which they are exposed [23,24]. Activity space is defined as “the local areas within which people move or travel in the course of their daily activities” [25]. Because activity space indicates where and how people have contact with their social and physical environments [26], it can be used as a measure of “people’s degree of mobility” [27]. The activity space of an individual can thus be used to explore the interaction between human activity and environmental context [28,29]. Conventionally, standard deviational ellipses, GPS trajectory buffers, minimum convex polygons, and kernel density surfaces have been used to represent human activity space [13,28,30]. Notwithstanding the improvements in the theory and methodology to assess environmental exposure and in the investigation of the contextual effects on health outcomes with activity space, substantial challenges remain.

Even with advanced activity space methods to assess individual environmental exposure, inconsistent findings of the environmental effects on health behaviors/outcomes have been observed in recent studies [31,32,33]. This suggests that the reliability of existing studies may be affected by the misspecification of the geographic context [34], which was recently articulated as the uncertain geographic context problem (UGCoP) by Kwan [14]. The UGCoP refers to the problem that findings of the effects of area-based environmental variables (e.g., land-use mix) on health outcomes or behavior (e.g., physical activity) can be affected by how contextual units are geographically delineated. The problem “arises because of the spatial uncertainty in the actual areas that exert contextual influences on the individuals being studied and the temporal uncertainty in the timing and duration in which individuals experienced these contextual influences” [35]. Existing activity space methods have limitations that may compromise their ability to mitigate the UGCoP both spatially and temporally. From the perspective of spatial uncertainty, conventional methods ignore the accessibility of different locations in the study area and thus may include locations that may not be accessible to people. Moreover, arbitrary cut-off distances are often used for delineating activity space. Temporally, the duration of environmental exposure is treated merely as the multiplier of exposure while individuals’ interactions with space during particular periods of time (the more time spent at the location, the more familiar with the sounding area) is not considered. 

This paper proposes the context-based crystal-growth (CCG) activity space as an innovative method for generating individual activity space based on both GPS tracking and environmental context. To mitigate the UGCoP, portable GPS devices are utilized to trace human movement accurately, and advanced GIS methods are used to relate these data to high-resolution data of relevant environmental contexts [14,36]. The integration of GPS and GIS provides a powerful means for examining the relationships between environmental contexts and health outcomes [22,37]. In contrast to other existing methods, and in order to address spatial uncertainty, activity space is generated considering not only people’s actual daily activity patterns based on GPS tracks but also the environmental contexts that either constrain or encourage people’s daily activity. Instead of using arbitrary cut-off distance, activity space is delineated based on the features of individual movement patterns. To mitigate temporal uncertainty, the duration of the environmental context in which individuals experienced and with which individuals interact are taken into account by abstracting the core areas of their daily activities. The size of activity space is based on the accumulated time an individual spent at the location (the more time a person spent there, the larger the activity space). To the best of our knowledge, this is the first study to introduce the context-based crystal-growth method and consider both people’s daily activity patterns and environmental context in activity space and environmental health research. The results indicate that the proposed new method generates more reasonable activity space and more accurate exposure assessment when compared to other existing methods. It can help mitigate the UGCoP spatially and temporally in environmental exposure measures. The accurate assessment of environmental exposures sheds light on the investigation of environmental effects in the field of epidemiology and health geography. The method is a new tool for activity space delineation and can be used for exploring the relationships among human movement patterns, environmental context, and health outcomes.

## 2. Methodology

**Figure 1:**
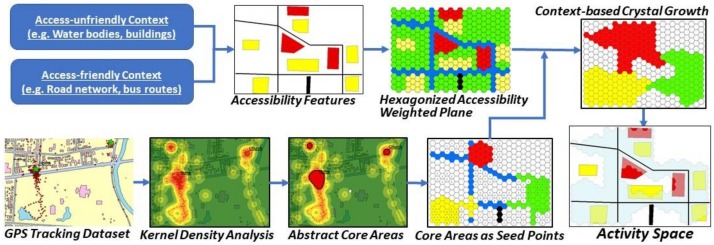
Workflow of the context-based crystal-growth activity space method.

This research proposes an innovative method for delineating individual activity space based on GPS tracking data, accessibility-related contextual data, and a crystal-growth algorithm. Due to the capability of incorporating GPS tracking and contextual data, as well as the flexibility to adaptively adjust the activity space based on the context of accessibility, this method is suitable for generating activity space while mitigating the UGCoP. In this method, accessibility-related contexts are incorporated into weighted planes, in which space is tiled into fine regular-grid cells (e.g., hexagonal cells). The weighted planes use hexagon grids to achieve higher accuracy in representing the spatial features of the land surface and minimize orientation bias and sampling bias from edge effects. The method is also capable of handling different transportation modes (e.g., walking, driving, taking the bus or train) while generating the activity space. Two accessibility-weighted planes are generated for public transport users and private transport users, respectively, considering the different effects of context for various groups of residents. Based on the weighted planes, space is delineated by the growth of cells from one or more seed points to neighbor cells through a sequence of growth cycles. The crystal-growth method is suitable for this study because the growth speed of each cell can be dynamically adjusted according to the accessibility-weighted planes, and the growth extent can be feasibly defined based on travel time. Figure 1 illustrates the workflow of the proposed context-based crystal-growth method. As people’s frequently visited locations are essential for understanding their daily activity, these places are considered as the core areas of their activity space, as identified by the kernel density analysis of an individual’s 7-day GPS trajectories. Thus, the activity space is grown from the core areas, and they will grow to their neighbor cells cycle by cycle. The crystal-growth process is either constrained or encouraged based on the accessibility-weighted planes. The merging of the crystal-growth space of all core areas generates the activity space.

### 2.1. GPS Tracking and Context Data

The individual GPS tracking dataset used in this research was collected as part of a larger study that examines the relationship among the exposure to environmental stressors, neighborhood quality, and individual health in Chicago. The larger study seeks to understand how the neighborhoods people live in and visit in their daily life affect their health and wellbeing. It focuses on the noise and air quality that people are exposed to in their daily life—not just at their residence, but also while they undertake their daily activities at other locations (e.g., travel to work, shopping, or running errands). The data were collected from October to December 2017 in the Chicago metropolitan area using surveys, GPS-equipped mobile phones, and portable noise and air pollutant sensors. The GPS tracking dataset is not recorded in even temporal duration but somewhat random over time depending on participants’ movement for prolonging battery life. To be specific, if a subject moves more than 1 m from the previous record within 3 s, a tracking point will be recorded by the tracking device. If the subject does not move more than 1 m from the last record in 3 s, a new tracking point is still recorded. In the GPS tracking dataset, each subject was tracked with GPS-equipped mobile phones for seven days. Because most people have highly routinized daily activities [38], 7-day continuous activity tracking, which covers both weekdays and weekends, can capture most of the participants’ weekly routine activities and is typically used for activity space research [38,39,40,41,42,43,44]. Consistent with previous studies, GPS tracks for 7 consecutive days were used to generate individual activity space in this study. 

To compare the activity space generated by different methods, as some of them are sensitive to participants’ movement patterns, four representative participants are selected from the dataset, as their movement trajectories have very different patterns (the spatial arrangement of the GPS points). The movement trajectories of Person A show a compact clustered pattern, those of Person B exhibit a modest clustered pattern, those of Person C display a one-directional pattern, and those of Person D present a multi-directional pattern. Note that due to the Institutional Review Board’s (IRB) requirements for protecting data confidentiality and participants’ privacy, we can describe the patterns but cannot include these maps in this paper.

Further, the activity spaces are used to assess environmental exposure with the whole dataset. The exposure to physical-activity-friendly contexts is used as a proxy for the comparison of activity space methods. Physical activity has been intensively studied in environmental health research [45,46,47] because it is highly related to many chronic diseases, such as type-II diabetes, obesity and cardiovascular diseases [8,48,49,50,51]. Although the results are inconsistent, most previous studies have observed a positive association between physical-activity-friendly environments and the level of physical activity [1,44,52,53,54,55,56,57]; the effectiveness of activity space for environmental exposure assessment can be evaluated by examining whether the association between exposure to the context and physical activity is captured. Physical-activity-friendly contexts include green spaces, blue spaces and other leisure facilities such as urban parks, playgrounds, swimming pools, and sports centers. The level of physical activity, which was reported by participants in the questionnaire before the GPS tracking, is measured by the number of days over a typical week in which a participant is physically active for a total of at least 30 min. There are 31 participants whose GPS tracking data in the dataset are used for such evaluation. The sociodemographic characteristics of these participants are shown in Table 1. The physical-activity-friendly contexts are just an example used to illustrate the usefulness of the proposed activity space method for environmental exposure assessment, which can also be applied to examine the effects of other environmental influences on other kinds of health behaviors or outcomes. For example, to explore the environmental influences on people’s body weight, we can include the availability of different types of food outlets or shops (e.g., fast-food outlets that sell unhealthy foods and supermarkets that provide more selections of healthy foods. To examine people’s exposure to air pollution, we can include traffic-related sources (e.g., highways) and various point sources (e.g., industrial plants; oil refineries) as environmental influences.

To generate a consistent and consecutive time series for the GPS trajectories, data cleaning and interpolation of GPS points was conducted so that there is one GPS tracking point each second for every participant for the 7-day tracking period. A Python program is developed to test every consecutive pair of GPS records and calculate the time difference. If the time difference between two consecutive GPS records is N seconds, which is longer than 1 s and shorter than 1800 s (half hour), linear interpolation in both spatial and temporal dimension is performed between the GPS records so that N-1 more records are inserted. After the interpolation process, each participant has one GPS tracking point for every second for the entire tracking period.

The environmental context data for this study were derived from a comprehensive digital geographic database of Chicago from the Chicago Data Portal as well as the volunteered geographic information website of OpenStreetMap. This includes the geographic location and footprint of buildings, water bodies, woods, restricted area (e.g., airport), and ground railways, which can be considered as barrier factors for accessibility; while the various levels of road networks, walkable areas, public transport routes are considered as the access friendly context that encourages accessibility. These contextual data are used to generate the two context-based hexagonal accessibility-weighted planes.

### 2.2. Core Areas of Daily Activities

People’s frequently-visited locations are crucial for understanding their daily activity and the delineation of their activity space. Therefore, these locations (core areas) are first abstracted from the GPS trajectories using kernel density analysis. The results of the kernel density analysis of the GPS trajectories are not distributed normally and skewed to the low-density values, so the geometric interval classification is a suitable method for classifying the density values by minimizing the sum of squares of the number of elements in each class. The algorithm creates geometric intervals to ensure that each class range has approximately the same number of values while keeping the change between intervals consistent [58]. The core areas are abstracted from the results of the kernel density analysis as the collection of cells with density value larger than the 3/4 cut point of the geometric interval classification of all non-zero values. These core areas will be used as the seed points in the following crystal-growth delineation. Further, the number of the growth cycle of each core area is weighted based on the accumulated time the individual spent at these locations. The more time a person spent there, the more cycles the activity space grows. The crystal-growth cycle of each core area is calculated based on the normalized sojourn time (NST) at each core area. For instance, one individual spent 600 min daily at home on average, so the normalized sojourn time at home ($NST_{h}$) is 600. The growth extent (GE) is dependent on the NST. We assume the growth extent from home location is $GE_{h}$, so the $GE_{i}$ of other core areas can be calculated based on the following formula:

(1) $$a=\sqrt[{GE_{h}}]{NST_{h}}$$

(2) $$GE_{i}=\log_{a}NST_{i}$$

where $GE_{i}$ is the growth extent of seed point i in minutes, $NST_{i}$ is the normalized sojourn time at core area i, $NST_{h}$ is the normalized sojourn time at home, $GE_{h}$ is the growth extent from the home location. 

As the only parameter for defining the growth extent of all the core areas, the $GE_{h}$ can be fixed universally for all subjects, and it can also be determined based on personal mobility. For the purpose of illustration, this study uses 10-mins’ travel as the universal value of $GE_{h}$. Although the $GE_{h}$ could be different for people with different mobility, 10-mins’ travel distance is a reasonable assumption that people are familiar with the environment and activity opportunities of the areas around the home, and it is highly possible that people choose to undertake the daily activity and are exposed to the context in this area.

### 2.3. Context-Based Hexagonal Accessibility-Weighted Planes

The accessibility-weighted plane, as a representation of the accessibility-friendliness of the environmental context, considers the effect of many kinds of environmental contexts (e.g., rivers as barriers, roads as thoroughfares) on the accessibility of the study area. The context of buildings, water bodies, woods, restricted areas, ground railways, road networks, public transport routes (metro and bus) and walkable areas are critical factors for the accessibility-weighted plane. Further, considering the different effects of environmental contexts for various groups of people (e.g., the expressway is considered as a thoroughfare for car users, while it is a barrier for public transport users), two context-based hexagonal accessibility-weighted planes are generated respectively for private transport users and public transport users.

For private transport users, on the one hand, among all these contexts, buildings, water bodies, woods, restricted areas (e.g., airport, private land) and ground railways are considered as barrier factors that are normally hard to trespass by people. On the other hand, various levels of road networks and walkable areas are accessibility-friendly context since they increase the general approachability of various sites. Road networks are further classified into expressways, primary roads, secondary roads and tertiary roads with various travel speeds. On the accessibility-weighted plane, as illustrated in Table 2, seed point cells are assigned a value of 100. The barrier cells (e.g., restricted areas and water bodies) are designated a value from 10 to 14 with a growth speed of 0. The transport network cells are assigned a value of 21 to 24, and the growth speed varies with their average travel speed. For pedestrian trails and walkable areas, cell values of 30 and 31 are assigned, respectively, and the growth speed is 1 cell per cycle (3 miles/h).

In contrast to private transport users, for public transport users, expressways, buildings, water bodies, woods, restricted areas and ground railways are considered as barriers, while metro routes and bus routes are considered as thoroughfares with higher accessibility. On the weighted plane for public transport users, all kinds of local road (including primary roads, secondary roads, tertiary roads, and pedestrian trails) are considered as walkable area only, since they do not have cars to drive on them. As listed in Table 3, local roads and walkable areas are assigned a value of 30 and 31, with a growth speed of 1 cell per cycle. Bus routes have a growth speed of 3 cells per cycle with a cell value of 23, while metro routes have a growth speed of 6 cells per cycle with a cell value of 21. Metro stations are also marked in the weighted plane (cell value: 22; growth speed: 1), since citizens can only get on or get off the metro system at stations. On the contrary, bus stations are not considered, since buses stop frequently and the distance between bus stations are only about 100 to 200 m in Chicago.

**Figure 2:**
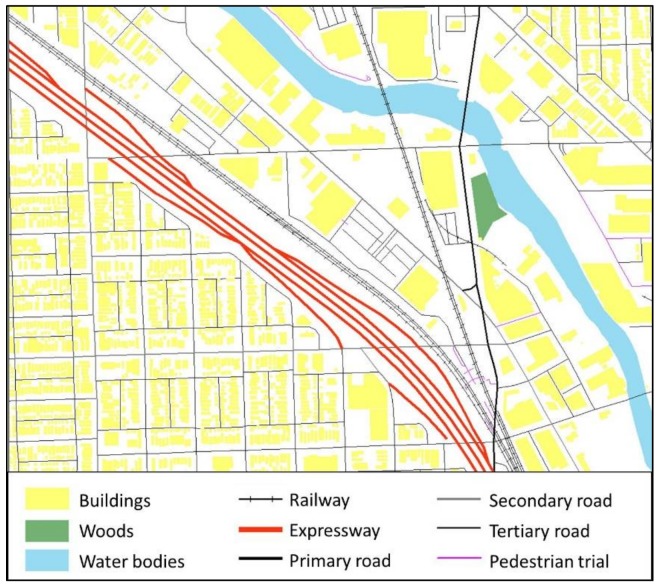
Accessibility-related environmental contexts.

**Figure 3:**
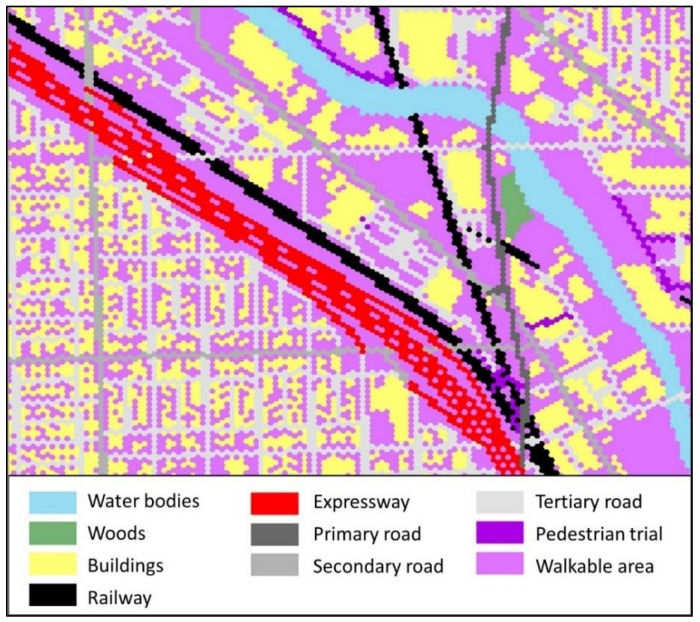
Hexagonal accessibility-weighted plane for private transport users.

**Figure 4:**
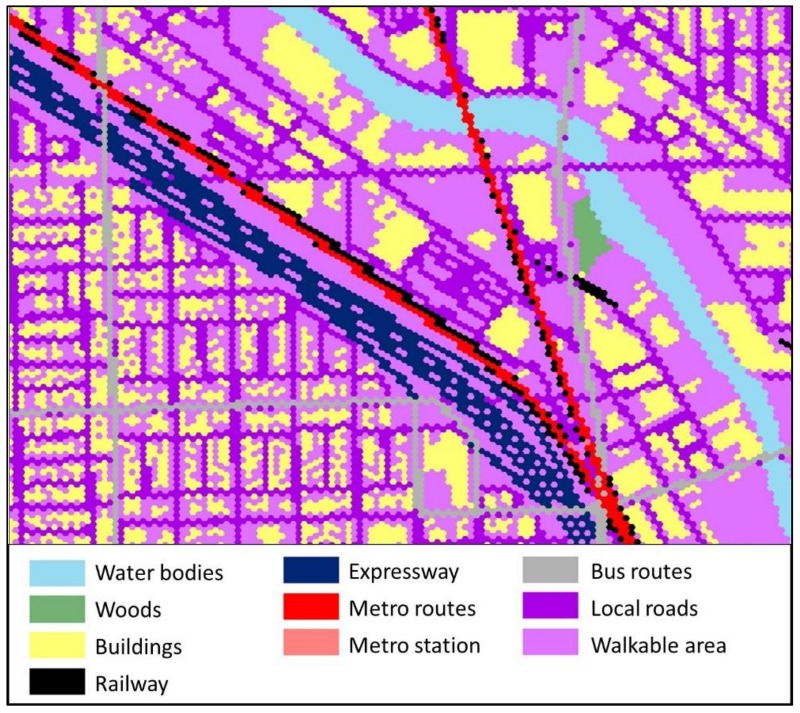
Hexagonal accessibility-weighted plane for public transport users.

**Figure 5:**
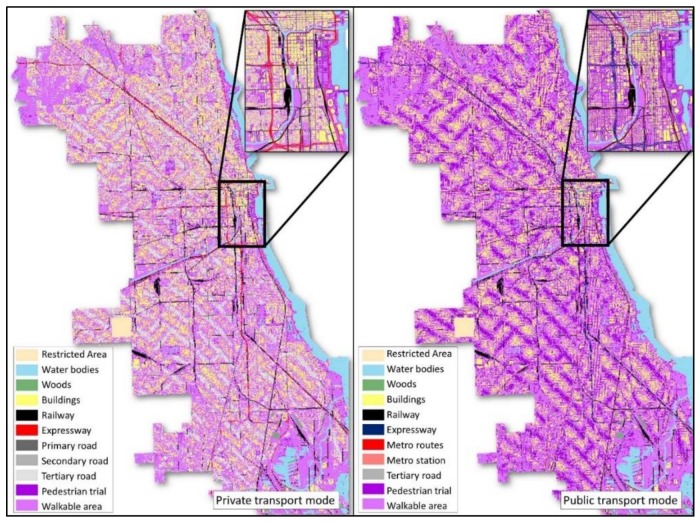
The context-based hexagonal accessibility-weighted plane of Chicago.

The geographical location and footprint of the environmental contexts are utilized to generate the hexagon-grid-based accessibility-weighted plane. The hexagonal grid, different from the raster grid, tiles the land surface with regularly sized hexagonal cells. They are the most compact regular polygons that can fill the land surface [59]. Hexagonal cells are closer in shape to circles [60,61], and they have only one kind of neighbor cells that share the same edge. Further, the distance to the centroid of a cell from the six neighboring cells is the same. The hexagon grid can achieve high accuracy in representing the spatial features of land surface from the perspective of spatial analysis [60], and it reduces the complexity in defining neighbor cells when compared to the raster grid. Thus, it is suitable for the crystal-growth algorithms used in this study. Figure 2 illustrates the environmental contexts of a neighborhood in Chicago, and the generated hexagonal accessibility-weighted plane for private transport users (see Figure 3) and public transport users (see Figure 4). The hexagonal cells in the weighted planes have a fine resolution of 10 × 10 m to ensure the accuracy of the calculation, and were generated using ArcMap. The context-based hexagonal accessibility-weighted planes of Chicago are shown in Figure 5; there are about 6 million hexagonal cells for each weighted plane that covers the city.

### 2.4. Hexagon-Grid Crystal-Growth Activity Space

The crystal-growth method is a tool for space partitioning and was used for Voronoi diagrams [62], spatial delineation [63], and spatial optimization [64]. The method is suitable for this study because the growth speed of each cell could be adjusted based on the context of accessibility. Since the context of accessibility is one of the critical factors for accurately generating activity space, which is rarely considered in previous research, this study utilizes the crystal-growth algorithm based on the hexagon-grid accessibility-weighted planes. 

**Figure 6:**
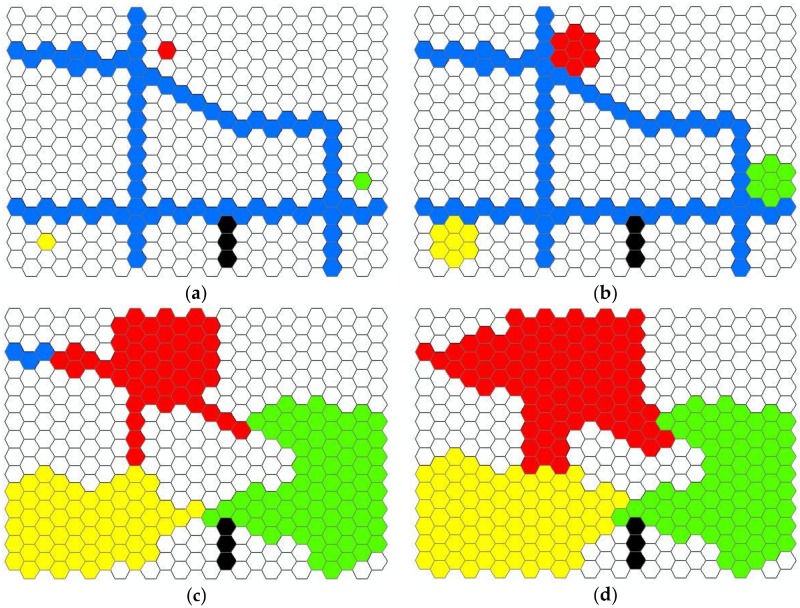
Illustration of the context-based crystal-growth activity space method. (**a**) The initial status with three seed cells; (**b**) crystal growth after one cycle; (**c**) crystal growth after several cycles; (**d**) the final result of crystal-growth. The red, yellow and green cells are three seed cells. Blue cells represent transport network, while black cells illustrate the physical barriers. The other white cells imply the walkable area.

Figure 6 illustrates this crystal-growth algorithm. In the method, the activity space is grown from all seed point cells (the location of the core areas), the service area of each seed point cell will grow to their neighbor cells cycle by cycle. Because the weighted planes simulate the road network and other physical barriers (Figure 6a), the growth speed of each location can be adjusted in real time based on the attributes of the cells. For instance, roads speed up growth, while rivers or lakes block the growth. For illustration, the road network is rendered as blue cells, while black cells represent barriers in the figure. As illustrated, crystal-growth starts from the seed point cells to their six immediate neighbor cells, and the growth continues cycle by cycle (Figure 6a,b illustrates one crystal-growth cycle). In contrast to other cells, the growth speed of transport network cells is faster due to the higher accessibility they enable (Figure 6b,c). Growth is constrained by natural barrier cells because it is usually challenging to travel through barriers such as rivers and lakes (Figure 6c,d). The growth of each seed point will stop when its maximum growth extent reached. The merging of the crystal-growth area of all core areas generates the final activity space.

According to the generation method of the context-based crystal-growth method discussed above, activity space is generated according to the following growth rules. (1) Based on participants’ travel mode, choose the context-based hexagonal accessibility-weighted plane accordingly for either private transport users or public transport users. (2) The crystal-growth starts from all the seed points (core area cells) separately. Seed point cells are the hexagon cells that intersect with the locations of the centroids of core areas. (3) For each crystal-growth cycle, if a neighbor cell is a barrier cell or a cell that has already been labeled as a grown area, it will be skipped. Otherwise, the neighbor cell will be marked as the grown area. (4) The crystal-growth speed of each cell is determined by the cell value, which was defined on the accessibility-weighted planes. (5) The crystal-growth will finish if the growth of all the seed cells reached their maximum growth extent. (6) The final activity space is generated by merging all the crystal-growth areas.

### 2.5. Other Existing Activity Space Methods

To compare the proposed context-based crystal-growth (CCG) activity space method with other existing methods, four commonly used delineations of activity space are implemented with the same GPS dataset, including GPS trajectory buffers (GTB), standard deviational ellipses (SDE), kernel density surfaces (KDS) and minimum convex polygons (MCP). GTB was created for each selected participant by covering the subject’s GPS trajectories with a 200-m buffer area, which covers all the locations that a participant visited or passed by during the study period. SDE is a commonly used method for delineating individual activity space. The SDE captures the geographic distribution and directional trend of all activity locations. The ellipse was obtained based on one or two standard deviations of the distances between each point and the transformed mean center along the rotated major and minor axes of the point set. Since past studies have used either one standard deviation SDE (SDE1) or two standard deviations SDE (SDE2), we derived both in this study for comparative purposes. KDS is a density surface derived from the activity locations and an associated weight using a kernel function and a predetermined search radius. In this study, the KDS was generated based on the duration spent at each GPS point as the weight on a raster layer at the spatial resolution of 10 × 10 m and search radius of 1000 m. MCP for a subject is the smallest convex polygon that contains all of the person’s GPS tracking points. It represents the smallest area that includes all activity destinations of a participant. 

## 3. Results

### 3.1. Context-Based Crystal-Growth Activity Space

**Figure 7:**
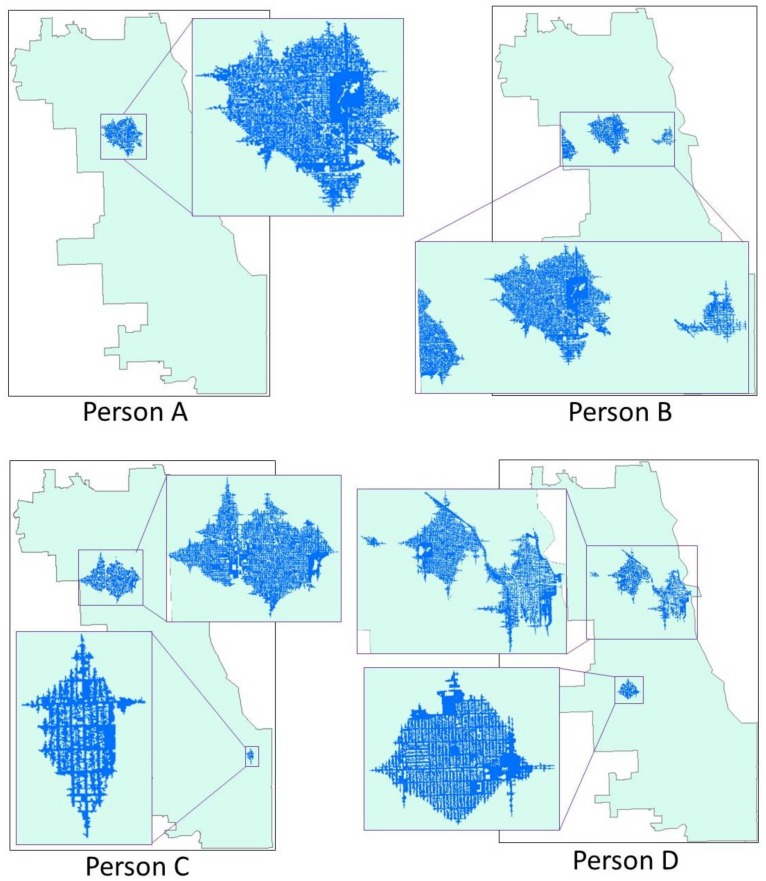
The context-based crystal-growth activity spaces of the four representative participants.

The CCG activity spaces delineated based on the four selected participants are illustrated in Figure 7. For person A, whose daily activity is highly concentrated around the home location, it shows a compact clustered pattern. Based on the collected demographic characteristics, this person is a retired female in her 60s, who spends most of her time at home and only visits the public library regularly. Only two core areas are detected, and they are close to each other. Thus, the CCG activity space is a grown area centered at these two locations within the travel distance of 10 min from home (one of the core areas) and the corresponding travel distance (calculated based on the normalized sojourn time) from the other core area (library). Because the growth is based on the accessibility-weighted plane, the activity space protrudes along major roads due to their higher accessibility than other contexts. There are several hollow areas in the activity space, which are barrier contexts, such as water bodies and private houses, that could not be easily trespassed. In contrast to the compact clustered pattern, person B presents a modest clustered pattern. Although the movement is also clustered around the home, this person has more activity locations and travels much further than person A to undertake daily activities. According to the participant’s profile, this subject is an unemployed male in his 40s. He visits his mother’s home frequently in the west of the city about half-hours’ drive from home. He also visits the downtown area to visit doctors and friends. Not surprisingly, three core areas are identified, and his CCG activity space is composed of three separate grown areas centered at home, mother’s house and downtown. The largest sub-area is the one centered at home (in the middle of the map) based on the fact that he spends the largest amount of time at home. He also spends much time at his mother’s house, and the sub-area should be larger than the current form, which is truncated due to edge effects. His mother’s home is close to the boundary of the study area, and thus only part of the sub-area is captured due to the lack of contextual information outside the border of the study area. Since the time he spends in the downtown area is much less when compared to the time spends in the other two core areas, this sub-area is much smaller. With a one-directional pattern, the CCG activity space of person C is exhibited in Figure 7. This person is a middle-aged male. He does grocery shopping and other personal activities around the home neighborhood. That is probably the reason why many core areas are identified around his residential neighborhood. What’s more, with his close relatives living in the southeastern part of the city, he needs to drive there and visit them regularly, and that is why we find another core area there. Thus, his activity space has a significant part centered at home and another small portion in the southeastern part of the city. Finally, person D is a female adult, who has a full-time job at downtown. With considerable mobility and travel around the city for daily activities, this participant’s movement shows a multi-directional pattern. As shown in the figure, the two largest portions of her activity space are the ones around home and workplace, since a significant amount of time is spent at these two locations. Other small parts of her activity space are scattered around the city for different daily activities. As a married woman, she needs to take care of the family, which includes grocery shopping and other household-related activities. It is noted that even though she traveled to the further north of the city, no activity space is identified, because she only spends limited time there, and it is not recognized as a core area. It is reasonable to exclude this kind of location from the activity space if the subject only visits the place with limited time on a nonregular basis.

**Figure 8:**
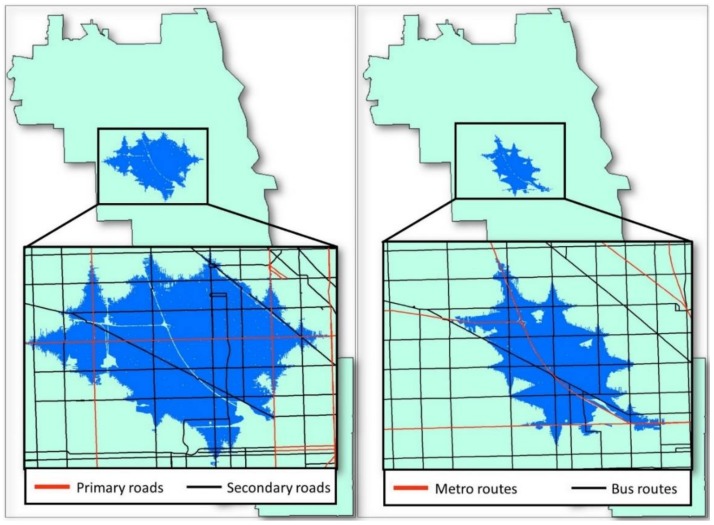
Crystal-growth activity space based on accessibility-weighted plane for private transport users (**left**) and public transport users (**right**).

To compare and illustrate the difference of CCG based on accessibility-weighted planes for private transport users and public transport users, respectively, as shown in Figure 8, both activity spaces are generated with the same core areas at the same locations. For private transport users, the activity space is grown and enlarged along primary and secondary roads. On the contrary, the public transport users relied heavily on the bus and metro systems, so the activity space extends along the bus and metro routes instead of the roads. It can be seen from the figure that the CCG activity space of public transport users is much smaller than that of private transport users.

### 3.2. Comparing the CCG Activity Spaces with Other Activity Spaces

#### 3.2.1. Comparing the Activity Spaces

**Figure 9:**
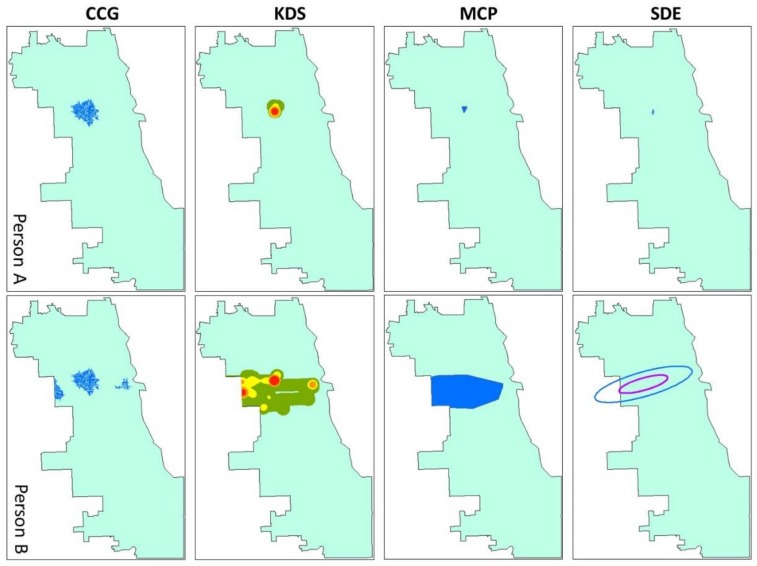

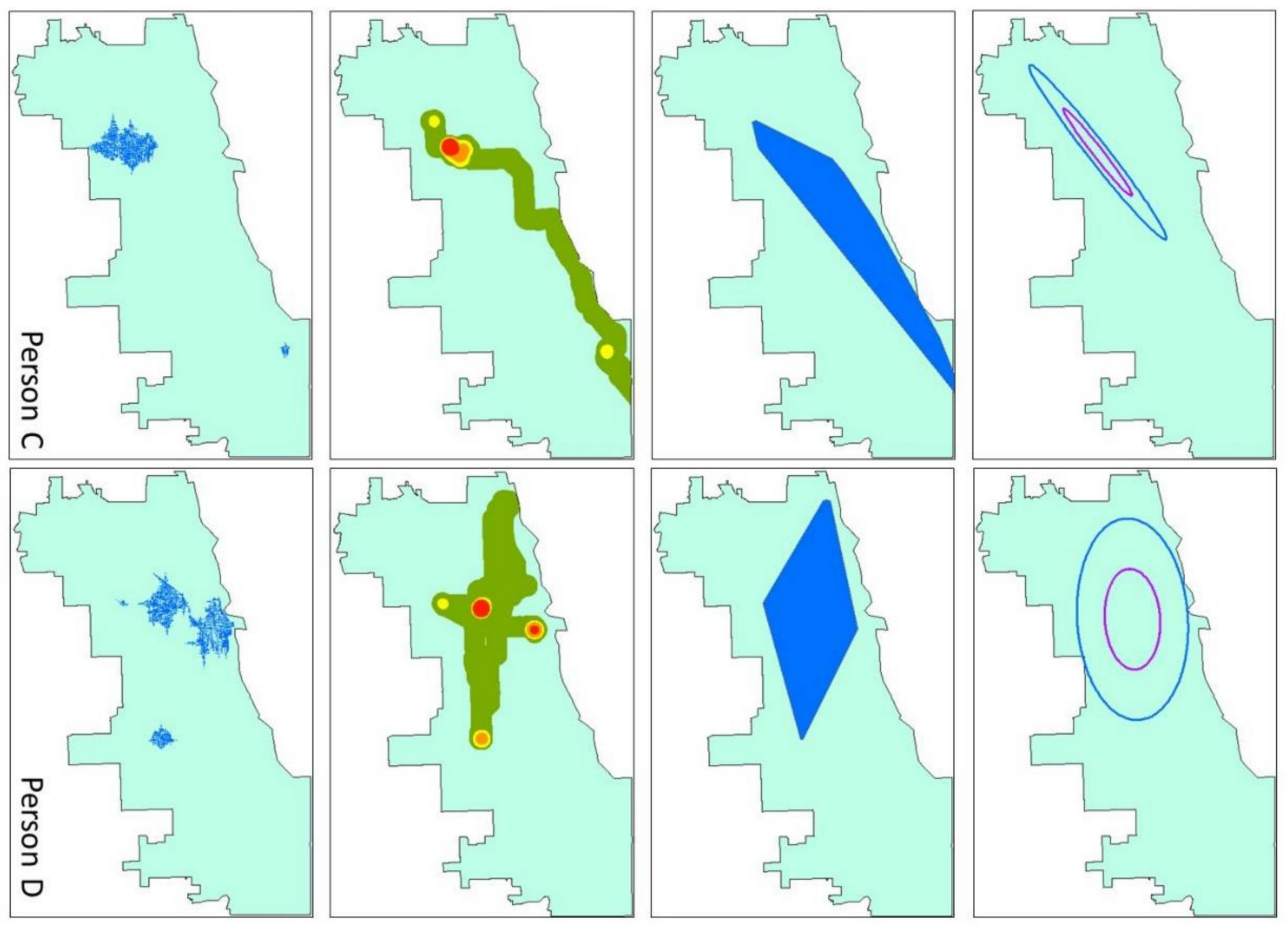
The four representative persons’ activity spaces generated by different methods.

The commonly used methods for activity space delineations are implemented with the four representative participants’ GPS tracking trajectory to compare with the proposed CCG method. The comparison is based on the geometric characteristics of the activity spaces, matching them with subjects’ daily activity, and visual interpretation. The activity spaces of the four participants are illustrated in Figure 9, and the comparison results are listed in Table 4. Note that the resulting maps of GTB, due to the risk of re-identification for the subjects, are not included in Figure 9 in order to protect their privacy. The activity spaces of person A are similar among the different methods because of the simplicity of the daily movement. Since person A has low mobility, and the GPS trajectories have a compact clustered pattern, all five methods generate activity spaces that are nearly circular, while focusing on the home location of the subject. The area of activity spaces generated by CCG (8.21 km^2^) and KDS (6.58 km^2^) are significantly larger than the area of activity spaces generated by the other two methods, while they cover almost all of the total GPS tracking points. For more complicated movement patterns (persons B, C, and D), the CCG is capable of generating multiple areas to portray individual activity space, whereas all the other methods only create a single continuous region. The area of the activity space of person B is larger than the one of person A. KDS (66.66 km^2^) and MCP (65.52 km^2^) have the largest area, while CCG (11.74 km^2^) has the smallest area. CCG activity spaces have the highest coverage of the total GPS tracking points except for the GTB, KDS, and MCP. For person C, the GTB, KDS, and MCP include not only the area for daily activity, but also the places along the travel trajectories between activity locations, leading to their larger areas. The directional pattern of the GPS trajectories makes the SDE highly compressed. Although the SDE does not include the places along the travel trajectory, it ignores the activity location in the southeastern part of the city and covers a lot of unrelated sites in the northwestern part. Still, the CCG activity spaces have the smallest size among all the activity spaces (18.76 km^2^) for person D. The dispersed GPS trajectories around the city make the area of the activity spaces generated with KDS, MCP, and SDE extremely huge, which covers many irrelevant city spaces. On average, the CCG has the smallest area (12.65 km^2^) among all the methods and the highest coverage (94.04%) of total GPS tracking points except for GTB, KDS, and MCP. Because of the nature of the techniques themselves, GTB, KDS, and MCP always cover all the GPS tracking points. On the contrary, CCG, and SDE include only the prominent parts of the points since they are more focused on the characteristics of the movement patterns instead of every single GPS point.

**Figure 10:**
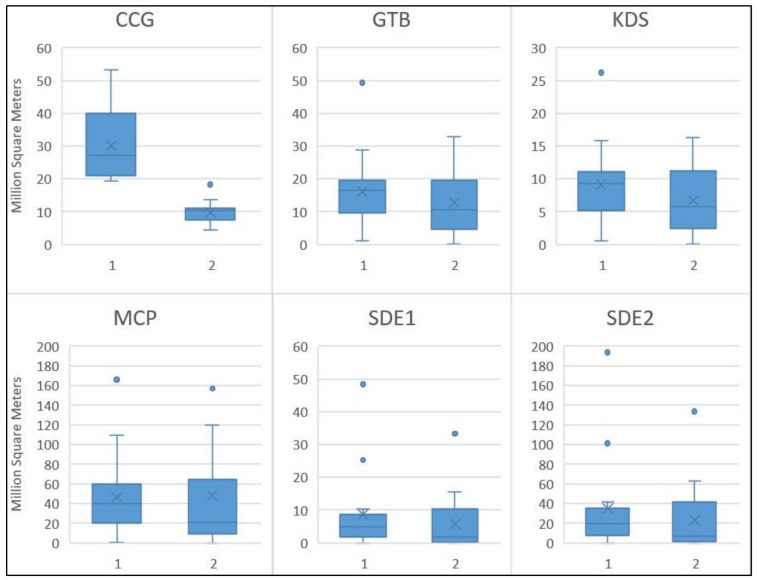
The differences in size of activity space for private transport (1) and public transport users (2).

For a more comprehensive comparative analysis, the activity spaces of the 31 participants in the dataset are also generated by CCG and the other five methods. Figure 10 illustrates the differences in the size of the activity spaces for private transport and public transport users. Considering the median size of the activity spaces, private transport users have larger activity spaces than those of public transport users for all the six methods; while the average size of the activity spaces of private transport users is still larger when compared to that of public transport users for all the methods except MCP. However, as shown in the boxplot in Figure 10, the difference is only significant for CCG.

#### 3.2.2. Comparing Physical-Activity-Friendly Contextual Exposures Measured by Different Activity Spaces

**Figure 11:**
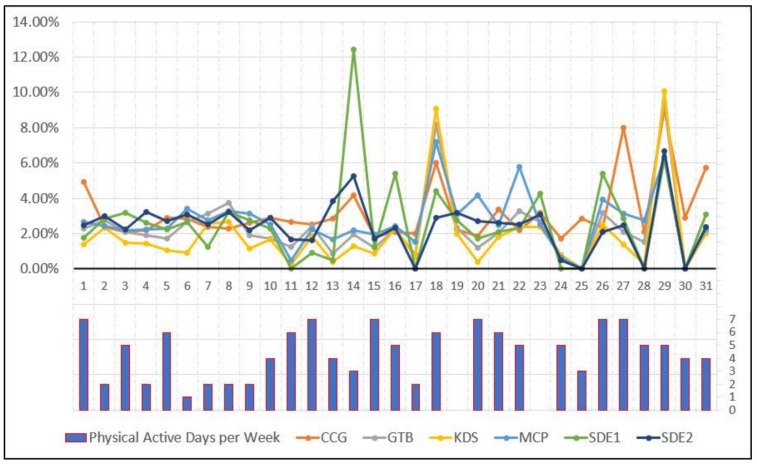
The physical-activity-friendly contextual exposures assessed by the different activity space methods and the physical active days per week for the 31 participants.

Physical-activity-friendly contextual exposures are assessed for the 31 participants by CCG activity spaces, as well as the other five methods. The assessment is based on the intersection between activity space polygon features and the physical-activity-friendly context polygon features. Contextual exposures are evaluated as the ratio of the area of the intersection polygons to the area of the activity space. In other words, they are calculated by the percentage of a participant’s activity space that is physical-activity-friendly. The higher the percentage value, the higher the assessed contextual exposure. The assessment results of the 31 participants are displayed in Figure 11. The top section illustrates the physical-activity-friendly contextual exposures assessed by the different activity space methods, while the bottom section depicts the number of days in which the participant is physically active for 30 min or more in a typical week. As indicated in the figure, CCG-assessed contextual exposures match the number of physically active days well. For instance, Participant 1 has a high physical activity level, while CCG is the only method that yielded high contextual exposure when compared to all other methods. For Participant 14, who has a medium level of physical activity, CCG estimated a reasonable level of contextual exposure, whereas SDE gave very high levels of exposure. Again, CCG is the only method that yielded moderate exposures, while all other methods gave extremely low values for Subject 25. 

**Figure 12:**
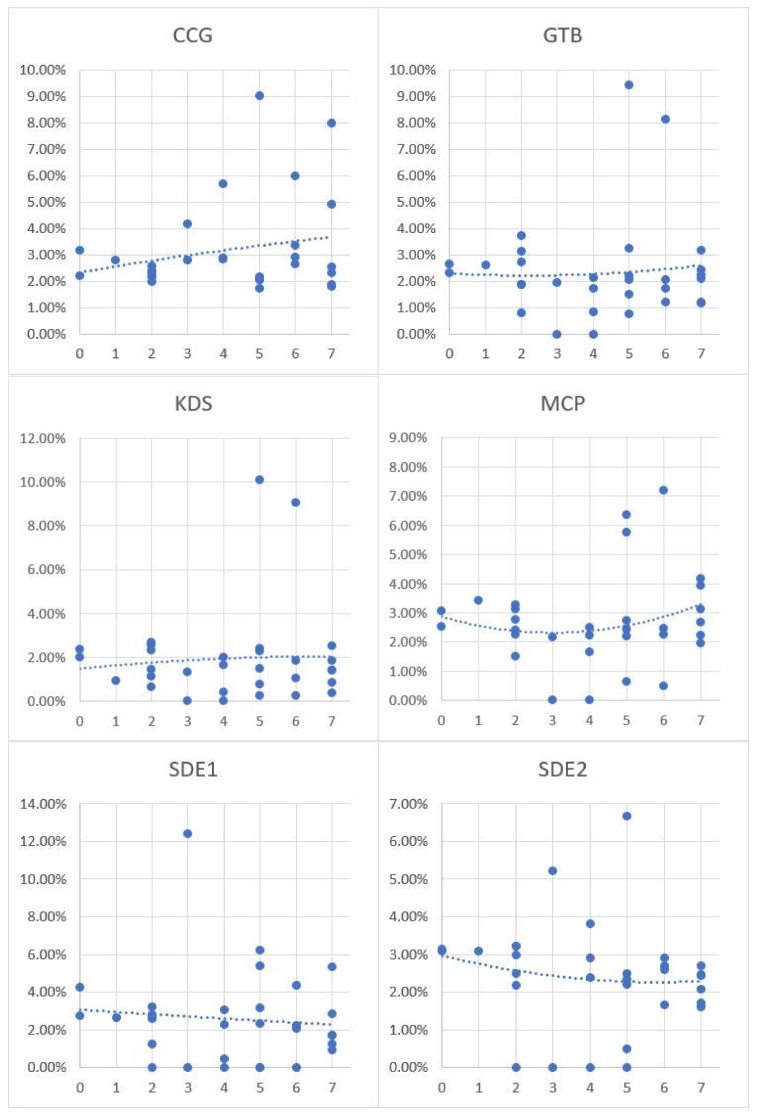
The correlation between physical-activity-friendly contextual exposures and physical activity level. (Vertical axis: the physical-activity-friendly contextual exposures level; horizontal axis: physical active days per week; trend lines are generated using the 2nd order polynomial fitting method.)

To further investigate the performance of the methods for discovering the relationship between physical-activity-friendly contextual exposures assessed by the different activity space methods and physical activity level is examined using a scatter plot with trend lines (Figure 12). As indicated in the figure, the contextual exposures measured by CCG and KDS show a positive correlation with physical activity level, while SDE1 and SDE2 reveal a negative association. For GTB and MCP, inconsistent relationships are observed. Although both CCG and KDS reveal the positive correlation between physical-activity-friendly contextual exposures and physical activity level, CCG has more robust results based on the trend lines and plot points in the figure.

## 4. Discussion

This study proposed an innovative method for delineating activity space for environmental exposure assessment based on GIS and GPS tracks data. By implementing the method with a GPS tracking dataset collected in Chicago, the proposed method generates more reasonable and reliable results when compared with other methods.

The proposed method focuses on movement pattern mining and core-area abstraction using the entire GPS trajectory patterns instead of individual GPS points. As listed in Table 4, the activity spaces derived with GTB, KDS, MCP are large and cover all the GPS tracking points. In these methods, each GPS point is indifferently considered as part of the activity space. The consequence of this is that the result includes not only the critical activity locations but also areas that participants passed by when traveling between activity locations. In contrast to other approaches, and by abstracting core areas based on GPS tracking data, the proposed method generates activity spaces based on the core areas that cover only the prominent parts of the points. All frequently visited activity destinations and more than 90% of the total GPS tracking records are covered in the CCG activity spaces. Further, the CCG can generate multiple areas to portrait individual activity space instead of a single continuous region, so the places that participants only passed by can be excluded. All other activity space techniques generate one activity space that unavoidably includes a large area of irrelevant space. This error can be exacerbated, as shown in Figure 9, when dealing with highly mobile subjects whose movement pattern is dispersed or strongly directional.

While other methods ignore the potential activity opportunities and environmental exposure around a person’s critical activity locations, which are also crucial factors for environmental exposure assessment, this study includes these potential areas to generate activity space by innovatively utilizing the crystal-growth algorithm. The idea is developed based on the fact that people are familiar with the environment and activity opportunities of the areas around their core activity locations (e.g., home, workplace). Even though they are not captured by their GPS trajectories during the tracking period, it is highly possible that people chose or will choose to undertake the daily activity and are or will be exposed to the contextual environments in these areas. For instance, in Figure 9, the CCG activity space of Person D contains a sub-area on the south of the city. According to the sojourn time spent in that area and the context of accessibility, an independent activity area is generated, which is centered at that core area and includes the potential activity opportunities. For other methods, GTB, KDS, and MCP activity spaces only include the actual GPS-tracked locations, while SDE doesn’t even cover that area, since it is so isolated from other activity locations.

By incorporating the hexagon-grid-based accessibility-weighted plane, this is the first study that takes into account the context of accessibility for activity space delineation. Accessibility is a critical but disregarded factor in previous research. Inaccessible areas in a study area may include private houses, water bodies and restricted areas (e.g., airport). Since people can only access and undertake daily activities at accessible locations, including inaccessible areas in the activity space introduces error for delineating people’s activity space as well as environmental exposure assessment. Further, the CCG method generates activity space from core areas based on specific travel time distance represented on the accessibility-weighted planes. Various levels of road networks and walkable areas are regarded as accessibility-friendly contexts with different travel speeds for private and public transport users, while people cannot trespass barrier contexts and need to bypass them, so more accurate results can be achieved by considering the context of accessibility. Additionally, the CCG method uses a hexagon grid for the weighted planes instead of the conventional raster grid to represent the context of accessibility more accurately.

The CCG method takes into account the ownership of automobiles, which is a critical factor that influences the size of people’s activity space but was widely ignored in previous studies. Although living in the same neighborhood, the transportation network and facilities have different effects on the accessibility of different groups of residents. For private transport users, road networks are treated as a high-accessibility context. Thus, their activity spaces grow and are enlarged along road networks. Notice that there is a primary road passing through the area from east to west in Figure 8, so the activity space extends in an east-west direction. While public transport users rely heavily on the bus and metro system, their activity space extends along the bus and metro routes. Further, different from private transport users who could utilize all the road networks effectively by driving their own cars, the public transport users can only increase their travel speed by taking buses or the metro. Although the bus routes are densely distributed in the area, the average travel speed is much lower than that of private cars. Not surprisingly, the CCG activity space of public transport users is much smaller than the ones of private transport users. By considering the different environmental effects of accessibility on different residents, as indicated in Figure 10, the CCG activity spaces of private transport users are significantly larger than those of public transport users, which highlights the fact that residents with automobiles have high accessibility and thus a large activity space. Whereas none of the other activity space methods consider the effects of automobile ownership on activity space, which introduces error in both the delineation of activity space and the assessment of environmental exposures. 

In this study, the exposure to physical-activity-friendly contexts is utilized as an example for comparing different activity space methods. Physical activity has been intensively investigated in past research, and it is highly related to obesity. The associations between physical-activity-friendly contexts and physical activity/obesity were found to be inconsistent and influenced by different delineations of activity space in environmental exposure assessment [66]. For instance, Zenk et al. [44] explored the environmental influences on dietary and physical activity through comparing the results generated by two different activity space methods (standard deviation ellipses and daily path areas), and inconsistent results were found. In Oliver et al.’s [67] study, the influence of land use on walking behaviors was examined by using 1-km circular and line-based road network buffers. The author found that the selection of activity space methods has considerable influences on the analytical results [67]. In addition, different kinds of activity space methods for built environment assessment were compared by analyzing their relationship with energy balance and obesity in other studies, e.g., [66,68]. These results indicate that activity space delineations have a significant influence on the observed associations between environmental exposures and health outcomes or behaviors [66,68]. Although the results are inconsistent, many past studies have observed the positive relationship between people’s exposures to physical-activity-friendly contexts and their physical activity level [44,52,53,54,55,56,57]. CCG is the only activity space method that discovered such positive association in this study. The comparative results in Figure 11 and Figure 12 support the idea that the proposed context-based crystal-growth activity space method performs better than other activity space methods in environmental exposure assessment. Although it is not feasible to use regression models to investigate the relationship between contextual exposures and participants’ physical activity due to the small sample size and the lack of other independent variables, this study obtained results that reasonably justify the usefulness and effectiveness of the proposed CCG activity space method for exposure assessment and environmental health studies. Further, although this study only implements the new method for assessing environmental influences on physical activity, it can be easily applied in other environmental health studies.

In summary, the CCG activity space method achieves a balance between the actual activities captured by GPS trajectories and the context-based potential activity opportunities around core areas, which are both crucial factors for environmental exposure assessment. According to the results, the SDE and MCP are too sensitive to the pattern of the GPS trajectories; they always include much irrelevant space that makes the activity space too large. Remote activity sites that are far from the other activity locations tend to be ignored by SDE. GTB is more focused on travel behavior and therefore ignores the important activity locations. With respect to KDS, similar to all other conventional methods, it disregards the context of accessibility. 

As discussed above, the existing activity space methods fail to mitigate the UGCoP. Spatially, the context of accessibility is ignored, and thus the activity spaces derived indifferently include many areas that may not be accessible. Furthermore, the effects of automobile ownership on activity space are ignored, therefore introducing uncertainty in the delineation of activity space. Moreover, arbitrary cut-off distance is used for the delineation of activity space, which adds error in the assessment of environmental exposures. Temporally, the duration in which individuals experienced environmental context is treated merely as a multiplier of exposure, while the interactions with space during the time (the more time spent at the location, the more familiar with the surrounding area) is overlooked. The CCG activity space method addresses these issues and delineates individual activity space more reasonably. It thus helps mitigate the UGCoP when assessing environmental exposure by people’s activity space.

Finally, this research has several limitations that need to be explored or addressed in future studies. First, this study implements the proposed activity space method based on the 7-day GPS trajectories of a small sample of participants and compares the results with other existing methods from the aspects of the geometric characteristics of the activity space, its correspondence with subjects’ daily activity, and visual interpretation. However, a larger dataset with the GPS trajectories of more subjects and further utilizing of the proposed technique for environmental exposure assessment are needed in future research to further evaluate and justify the method. Second, although the CCG is compared with other existing approaches based on GPS tracking data, there are also methods that don’t rely on GPS or GIS. For instance, studies that used map-based electronic questionnaires [38,69], mobility surveys [70] and activity space questionnaires [71] also yielded useful results. Although these qualitative methods based on self-reported information may include recall bias and not be geographically accurate, they can capture more background information about participants’ activities [38], such as the transportation modes and social interactions [72]. Thus, further study is needed to compare these qualitative methods or integrate them with CCG to generate more accurate activity space. Third, the final activity space is hexagon-grid based, so distance decay functions can be applied optionally to the results to generate a weighted activity space, in which the core areas have a weigh according to their NST values. The weights of other cells can be calculated with specific distance decay functions based on travel distance from the core areas. Fourth, as the only parameter when calculating the growth extent of all core areas, for the purpose of illustration, $GE_{h}$ is set to 10-mins’ travel distance for all subjects in this study. However, it can also be determined based on personal mobility (such as age and health condition) to increase accuracy. Fifth, the method is based on the assumption that people are familiar with the environment and activity opportunities of the areas around the core areas, and it is highly possible that people choose to undertake the daily activity and expose to the context in these areas. However, even if only rarely, it is possible that one person may spend a lot of time at one location but is still not familiar with the surrounding area. This problem could be addressed by cross-validation with an activity diary data in future studies.

## 5. Conclusions

This study proposed an innovative method for delineating activity space using individual GPS trajectories and a crystal-growth algorithm based on hexagon-grid accessibility-weighted planes. It generates a more reasonable activity space and captures people’s environmental exposures more accurately when compared to other methods. It is a new tool for activity space delineation that can be used for exploring the relationships between human movement patterns and environmental context as well as accessibility. It has considerable potential for making a groundbreaking contribution to the advancement of methods by introducing and developing a new analytical framework that allows the examination of activity space and individual environmental exposures while mitigating the UGCoP.

## Figures and Tables

**Table 1:** Sociodemographic characteristics of the participants in this study.

Sociodemographic Variables		Percentage
Gender	Male	61.3%
	Female	38.7%
Age	18–30	17.2%
	31–40	17.2%
	41–50	24.1%
	51–65	41.4%
Race	White	9.7%
	African American	41.9%
	Latino/Hispanic	41.9%
	Other	6.5%
Education	Elementary School	6.5%
	High School	58.1%
	College/University	32.3%
	Graduate School	3.2%
Marital Status	Married	17.9%
	Divorced	14.3%
	Single	67.9%
Annual Income	Less than $10,000	58.1%
	$10,000–$24,999	19.4%
	$25,000–$50,000	9.7%
	$50,000–$99,000	9.7%
	$100,000 or more	3.2%

**Table 2:** Cell attributes of the hexagonal accessibility-weighted plane for private transport users.

Context Type	Cell Value	Average Moving Velocity	Growth Speed (Cells/Cycle)
Out of Research Area	0	-	0
Restricted Area	10	-	0
Water Bodies	11	-	0
Woods	12	-	0
Buildings	13	-	0
Railway	14	-	0
Expressway	21	About 35 miles/h	12
Primary Road	22	About 25 miles/h	8
Secondary Road	23	About 15 miles/h	5
Tertiary Road	24	About 9 miles/h	3
Pedestrian Trail	30	About 3 miles/h	1
Walkable Area	31	About 3 miles/h	1
Seed Points	100	About 3 miles/h	1

**Table 3:** Cell attributes of the hexagonal accessibility-weighted plane for public transport users.

Context Type	Cell Value	Average Moving Velocity	Growth Speed (Cells/Cycle)
Out of Research Area	0	-	0
Restricted Area	10	-	0
Water Bodies	11	-	0
Woods	12	-	0
Buildings	13	-	0
Railway	14	-	0
Expressway	15	-	0
Metro Routes	21	About 18 miles/h	6
Metro Stations	22	About 3 miles/h	1
Bus Routes	23	About 9 miles/h	3
Local Roads	30	About 3 miles/h	1
Walkable Area	31	About 3 miles/h	1
Seed Points	100	About 3 miles/h	1

**Table 4:** Comparing the representative participants’ activity spaces generated by different methods.

		CCG	GTB	KDS	MCP	SDE1	SDE2
Person A	Area (km^2^)	8.21	0.91	6.58	0.52	0.0373	0.15
	%GTPC	100%	100%	100%	100%	93.28%	93.45%
Person B	Area (km^2^)	11.74	31.14	66.66	65.52	12.704	50.82
	%GTPC	87.97%	100%	100%	100%	76.47%	85.71%
Person C	Area (km^2^)	11.89	19.14	81.20	105.10	7.71	30.83
	%GTPC	96.60%	100%	100%	100%	82.04%	93.72%
Person D	Area (km^2^)	18.76	28.92	101.28	109.16	39.12	156.48
	%GTPC	91.69%	100%	100%	100%	75.69%	90.83%
Average	Area (km^2^)	12.65	20.03	63.93	70.08	14.89	59.57
	%GTPC	94.04%	100%	100%	100%	81.87%	90.93%

Note: the area of KDS is measured as the total area with positive density, which is consistent with Schonfelder and Axhausen [65]; %GTPC: percent of total GPS tracking points covered.
